# Seasonal Variation in Diurnal Rhythms of the Human Eye: Implications for Continuing Ocular Growth in Adolescents and Young Adults

**DOI:** 10.1167/iovs.63.11.20

**Published:** 2022-10-25

**Authors:** Nickolai G. Nilsen, Stuart J. Gilson, Hilde R. Pedersen, Lene A. Hagen, Kenneth Knoblauch, Rigmor C. Baraas

**Affiliations:** 1 National Centre for Optics, Vision and Eye Care, Faculty of Health and Social Sciences, University of South-Eastern Norway, Kongsberg, Norway; 2 Stem-Cell and Brain Research Institute, INSERM U1208, Bron, France; 3 Université de Lyon, Université Lyon I, Lyon, France

**Keywords:** circadian rhythm, melatonin, choroid, ocular growth, myopia

## Abstract

**Purpose:**

To investigate the diurnal rhythms in the human eye in winter and summer in southeast Norway (latitude 60°N).

**Methods:**

Eight measures (epochs) of intraocular pressure, ocular biometry, and optical coherence tomography were obtained from healthy participants (17–24 years of age) on a mid-winter's day (*n* = 35; 6 hours of daylight at solstice) and on a day the following summer (*n* = 24; 18 hours of daylight at solstice). Participants wore an activity monitor 7 days before measurements. The epochs were scheduled relative to the individual's habitual wake and sleep time: two in the day (morning and midday) and six in the evening (every hour until and 1 hour after sleep time). Saliva was collected for melatonin. A linear mixed-effects model was used to determine significant diurnal variations, and a sinusoid with a 24-hour period was fitted to the data with a nonlinear mixed-effects model to estimate rhythmic statistics.

**Results:**

All parameters underwent significant diurnal variation in winter and summer (*P* < 0.002). A 1-hour phase advance was observed for melatonin and ocular axial length in the summer (*P* < 0.001). The degree of change in axial length was associated with axial length phase advance (*R*^2^ = 0.81, *P* < 0.001) and choroidal thickening (*R*^2^ = 0.54, *P* < 0.001) in summer.

**Conclusions:**

Diurnal rhythms in ocular biometry appear to be synchronized with melatonin secretion in both winter and summer, revealing seasonal variation of diurnal rhythms in young adult eyes. The association between axial length and seasonal changes in the phase relationships between ocular parameters and melatonin suggests a between-individual variation in adaptation to seasonal changes in ocular diurnal rhythms.

Daylight is the strongest zeitgeber to entrain an individual's master circadian clock. Maintaining a regular diurnal rhythm (i.e., one that is of an approximately 24-hour period) is known to have general health benefits.^1^ The components and parameters in the eye also undergo diurnal rhythms.^2^^–^^6^ Experimental animal studies have shown that disruptions of ocular diurnal rhythms are implicated in accelerated ocular growth (axial elongation that leads to a more myopic refractive error).^7^^,^^8^ First, it has been reported that during normal growth there is a near-antiphase (9-hour) relationship between ocular axial length (AL) and choroidal thickness (ChT). In eyes undergoing stimulated growth, this shifts to an exact antiphase (12-hours) relationship during accelerated growth and an in-phase relationship during recovery from either form-deprived or lens-induced myopia.^9^^,^^10^ Second, both constant light and constant darkness have been associated with accelerated growth,^11^^,^^12^ implying that maintaining a balanced light/dark cycle is important for coordinated growth (axial elongation without a change in refractive error).^13^^,^^14^ In humans, it has been reported that myopic defocus reverses the phase relationship between AL and ChT,^15^ whereas hyperopic defocus leaves the phase relationship unaltered, with only short-term increases in AL and AL amplitude.^16^ In animal models, thinning versus thickening of the choroid has been associated with accelerated versus decelerated ocular axial elongation.^17^ Choroidal thickening has been linked to decreased scleral matrix remodeling,^10^^,^^18^ with regulation of ocular growth through prevention of mechanical stretching of the sclera.^17^^,^^18^ In humans, thinner choroids are reported to be associated with longer eyes.^7^

Daylight exposure, through outdoor activity during daylight hours, is the behavioral factor reported to have the strongest protective role against accelerated ocular growth.^19^^,^^20^ The mechanisms by which daylight regulates eye growth are not fully understood.^19^ Evidence from both human and animal studies show that light exposure, as well as imposed defocus, affect the ocular structures differently depending on time of day; light therapy has the largest effect on thickening the human choroid in the morning,^21^ whereas in chicks myopic defocus inhibits ocular growth more effectively in the evening than in the morning.^22^ These differential effects of the time of day raise the question of whether daylight exposure could be linked with improvements not only in overall bodily diurnal rhythm^1^ but also in ocular diurnal rhythm and subsequently the diurnal interrelationship among the different ocular structures.

Scandinavian countries seem to be defying the expected worldwide increase in the prevalence of myopia,^23^^–^^25^ with considerably lower myopia prevalence than that reported in southeast Asian countries, such as China.^26^ Scandinavia is located at the subarctic Northern Hemisphere where there are large seasonal variations of daylight length, intensity, and temperature.^27^ Even in southeast Norway, daylight duration can vary between 6 hours in the winter and 18 hours in the summer.^28^ Because of the low number of available daylight hours in the winter, the low myopia prevalence leads to a supposition of whether seasonal variations in daylight play a role in promoting coordinated growth.^23^ Slowing of eye growth and less myopia progression have been reported in the summer due to a higher number of available daylight hours.^29^^,^^30^ However, effects of seasonal variation of daylight on ocular diurnal rhythms, and its relation to eye growth have not yet been properly explored. To date, the only study looking at daily variations of AL and ChT across seasons was conducted in northeastern Australia, where there are minimal seasonal variations of daylight duration.^31^ If daylight plays a prominent role for maintaining healthy (normal growth regulation) phase relationships among ocular structures,^3^^–^^6^ we hypothesized that the availability of daylight hours in winter compared with summer should have a measurable effect on these phase relationships. Furthermore, the diurnal variations of the ocular structures (except for photoreceptors in the retina which are driven by intrinsic oscillators)^32^ might be synchronized to the master circadian clock (located in the suprachiasmatic nucleus), as measured by saliva melatonin secretion.^33^ With this in mind, we investigated diurnal variations of melatonin secretion and ocular structures in southeast Norway (latitude 60°N) in winter and the following summer in healthy high school and university students.

## Methods

### Recruitment

Thirty-five healthy young adults (19 females), 17 to 24 years of age, participated in this study. The study was approved by the Regional Committee for Medical and Health Research Ethics South East Norway and was carried out in accordance with the tenets of the Declaration of Helsinki. Informed consent was obtained from the individuals prior to participation. Baseline cycloplegic autorefraction (HRK-8000A; Huvitz Co., Ltd., Gyeonggi-do, Korea) was measured 20 minutes after instillation of topical 1% cyclopentolate hydrochloride (Minims single-use; Bausch + Lomb UK Ltd, Kingston upon Thames, UK). One drop of cyclopentolate was used if the participant's irides were blue to green and two drops if they were green to brown. The HRK-8000A incorporates an automatic three-axis eye tracker that optimally repositions the sensor to compensate for the participant's eye movements between measurements, intended to give high measurement repeatability. All participants had best-corrected distance visual acuity of ≤0.00 logMAR (TestChart 2000; Thomson Software Solutions, London, UK); anisometropia of <1.00 diopters (D); stereo acuity of ≤120 seconds of arc (TNO Stereotest; Laméris Ootech, Ede, Netherlands); and no errors on color vision tests, including the Ishihara, 24-plate edition (Kanehara Trading Inc., Tokyo, Japan) and Hardy–Rand–Rittler Pseudoisochromatic Plate Test, 4th edition (Richmond Products, Albuquerque, NM, USA). None of the participants had systemic or ocular disease, used melatonin supplements, or were diagnosed with sleep or mental disorders.

### Data Gathering Protocol, First Winter and Summer Measurements

**Figure 1.**
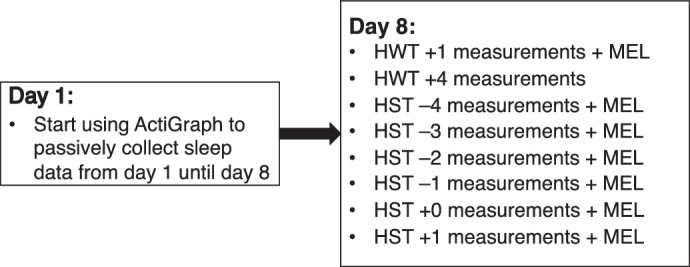
Sequence of measurements performed on the 2 days of data collection. The same protocol was used for both winter and summer seasons. In the figure, MEL indicates that a saliva sample was taken for melatonin analysis.

On day 1, self-reported habitual wake time (HWT) and habitual sleep time (HST) were recorded. The participants were given an ActiGraph GT3X wrist-mounted activity monitor (ActiGraph Corp., Pensacola, FL, USA) to wear for the subsequent 7 days and nights to objectively measure their habitual get-out-of-bed times and sleep-onset times (Fig. 1). On day 8, the participants returned to the lab for eight epochs of measurements. The first two epochs (HWT+1 and HWT+4) were 1 and 4 hours after the participant's self-reported wake times, respectively (the ActiGraph cannot measure wake times). The remaining six epochs were every hour from 4 hours before the participant's HST (as determined by the ActiGraph, using only weekday averages) until 1 hour after (HST–4 to HST+1), ensuring that all measurement epochs were aligned to the individual's chronotype. Participants did not sleep during the measurement period, including the HST+1 epoch.

Throughout each epoch, the participants remained seated on a wheeled chair to avoid the influence of postural changes on measurements.^34^^–^^36^ Thereafter the participants relaxed their accommodation by watching a movie binocularly at 5 meters for 15 minutes (accommodation washout),^16^ using their habitual distance correction if necessary. Each epoch entailed retinal and choroidal imaging by optical coherence tomography (OCT; SPECTRALIS OCT2 EDI; Heidelberg Engineering, Heidelberg, Germany); measurements of central corneal thickness, corneal radius, anterior chamber depth (ACD), crystalline lens thickness (LT), and AL (IOLMaster 700; Carl Zeiss Meditec AG, Jena, Germany); and intraocular pressure (IOP) (iCare; Tiolat Oy, Helsinki, Finland). The IOLMaster 700 is known to have high measurement repeatability.^37^ The recorded IOP measurements were the average of three sets where each consisted of six single measures for each eye. Saliva samples (Salivette saliva collection kit; Sarstedt, Nümbrecht, Germany) for analysis of melatonin (MEL) were collected during the last 5 minutes of the accommodation washout, at HWT+1 and at the six evening epochs. The saliva samples were immediately centrifuged at −4°C, 4400 rpm, for 5 minutes (Centrifuge 5702R; Eppendorf SE, Hamburg, Germany) and stored in a −25°C freezer. The samples were analyzed by VITAS Analytical Services (Oslo, Norway) using an ELISA kit (Bühlmann Laboratories, Schönenbuch, Switzerland).

In accordance with previously described guidelines, the participants did not consume or drink certain substances (such as NSAIDs, nicotine, bananas, chocolate) 36 hours prior to—or during—day 8 to avoid any undesirable effects on saliva samples or melatonin.^38^^,^^39^ Similarly, participants did not consume alcohol or drinks containing caffeine or artificial additives 24 hours prior to or during the experiment. None reported having traveled across more than two time zones within the month prior to the experiments. They were encouraged to wake and sleep as they normally would between day 1 and day 8. Ambient light levels, including the movie displayed on the television, were kept below 20 lux to avoid suppression of melatonin secretion^39^ or affecting the choroid.^40^

### Data Gathering Protocol, Second Winter Measurements

Ten of the participants (seven females), 19 to 24 years of age, returned for a third measurement of cycloplegic autorefraction and ocular biometry (same instruments and methods as described above) in the following winter between November and January.

### Winter and Summer Solstice

Experiments were conducted in the southeast of Norway (latitude 60°N, longitude 9°E) over a 2-year period. Participants were examined in either the 2018/2019 winter or the 2019/2020 winter and the following summer. The aim was to measure the participants as near as possible to both winter and summer solstices to observe the effects of daylight exposure at their most extreme. The winter solstice occurred on December 21, and participants were measured from 32 days to 4 days prior to this date (from 7 hours and 19 minutes down to 6 hours of available daylight). The summer solstice was June 21, and participants were measured from 39 days prior to the solstice and up to 5 days after (from 16 hours and 55 minutes up to 18 hours and 43 minutes of available daylight).^28^ From the 35 participants measured in the winter, 24 returned and completed the summer measurements. Although measurements were scheduled to each individual's habitual sleep pattern (Fig. 1), local clock time (UTC+1 during winter, UTC+2 during summer) was also recorded for each measurement. Henceforth, all clock times are reported in standard time (UTC+1).

### OCT Measurement Protocol and Segmentation Details

**Figure 2.**
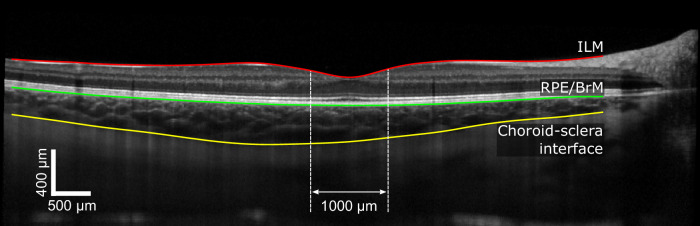
An OCT B-scan annotated with segmented retinal and choroidal layers. Retinal thickness and choroidal thickness were defined as the average area between the inner limiting membrane (ILM) and RPE layers and between the RPE and choroid layers, respectively, averaged over the central 1 mm and over both horizontal and vertical B-scans (horizontal shown).

The OCT scan protocol consisted of six radial scans centered on the fovea with 100 B-scans averaged at each orientation and with enhanced depth information enabled. The first measurement was used as the reference image for all subsequent epochs using the retinal tracking system of the instrument. A semiautomatic active contour method was used to segment the retinal and choroidal layers, as described previously.^41^^,^^42^ The lateral scale of the OCT scans was corrected for each individual's ocular biometry (from an IOLMaster 700 measurement at the same time point) using a four-surface schematic eye model.^43^^,^^44^ The anterior edge of the inner limiting membrane, posterior boundary retinal pigment epithelium (RPE)–Bruch's membrane band and the inner border of the sclera were segmented by two experienced operators.^45^^,^^46^ Only horizontal and vertical scans were used for analysis. Mean values were extracted for the central 1-mm retinal thickness (RT) and ChT (Fig. 2).

### Data Analysis

The statistical analysis was performed with R 3.6.3 (R Foundation for Statistical Computing, Vienna, Austria)^47^ with the nlme package.^48^ Parametric tests were used when the data were normally distributed; otherwise, a non-parametric alternative was used. Statistical significance level was set at α = 0.05. Spherical equivalent refraction (SER) was calculated as sphere + ½ cylinder. A random-effects ANOVA model was used to estimate within-session repeatability for the five autorefractor measures of cycloplegic SER, with a nested random-effects structure for “participant” and “eye” using the lme4 package.^37^^,^^49^ The within-session SD was estimated by profiling the likelihood.^50^ The mean of the five measures of SER were used for further analysis. Vitreous chamber depth (VCD) was calculated as AL – (ACD + LT + RT). The right eye was arbitrarily chosen for analysis, as there were no difference between OD and OS for any of the ocular biometry measures. Myopia was defined as SER ≤ −0.50 D, emmetropia as −0.50 D < SER < +0.50 D, and hyperopia as SER ≥ +0.50 D. Interrater reliability for segmentation of the choroid was assessed by calculating the intraclass correlation (ICC) with the irr package^51^ in R using a one-way model. ICC was 0.95 (95% confidence interval [CI], 0.929–0.965). The ActiGraph data were processed using ActiGraph ActiLife software. Wear time validation was checked with the Choi algorithm,^52^ and sleep periods were scored with the Sadeh algorithm as recommended for the age group included in this study.^53^ The ActiGraph GT3X has been found to be comparable to polysomnography.^54^

The epochal data were modeled with a sinusoid with a fixed 24-hour period^55^:

(1) $$y\left(t\right)=M+A\sin\left(2\pi \left(t+\varphi \right)/24\right)$$

using a nonlinear mixed-effects (NLME) model, where *M*, *A*, and φ are the midline estimating statistic of rhythm (MESOR), amplitude, and phase of the sine wave, respectively; *y*(*t*) was the measured value at epoch hour (*t*) relative to the participant's HST (e.g., *t* = –3 represents the HST–3 measurement). Acrophase is the timing of changes of amplitude within a 24-hour period and indicates how melatonin and ocular parameters co-vary in amplitude with each other throughout a single day. MESOR is the average magnitude in a 24-hour period and can be used to represent co-variation across seasons. For melatonin only, the sinusoid was fitted to the logarithm of the melatonin measurement, log(*y*(*t*)), which better captures the profile of melatonin secretion throughout the day.^56^ In order to capture the onset of melatonin secretion, dim light melatonin onset (DLMO), with a threshold of 3 pg/mL,^38^^,^^57^^,^^58^ was derived for each individual by solving Equation 2 and substituting log(*y*(*t*)) with log(3):

(2) $$\mathrm{DLMO}=\frac{24\mathrm{si}n^{-1}\left(\frac{\log\left(3\right)-M}{A}\right)}{2\pi }-\varphi$$

A linear mixed-effects model with epoch as a repeated factor and SER group as a fixed effect was used to identify significant diurnal variations in the ocular parameters and MEL.^31^ Repeated-measures ANOVA was used to study the differences between SER groups across seasons for different ocular parameters and MEL.

## Results

Table 1 shows the winter baseline measurements for the 35 participants. The within-session SD for the Huvitz HRK-8000A for these 35 participants was estimated to be 0.056 D (95% CI obtained by profiling the likelihood, 0.052–0.061). This compares favorably with the median value (0.054 D; interquartile range, 0.042–0.073) of the values estimated from each individual using *t*-statistics. The maximum of these individual values gives a worst case of ±0.176 D. Thirteen participants had hyperopia, 11 had emmetropia, and 11 had myopia. Mean AL was 23.83 mm, and mean cycloplegic SER was –0.50 D. Myopes were significantly older than hyperopes (*P* = 0.010), and there was a significant difference in AL and cycloplegic SER among the SER groups (all *P* < 0.001; Supplementary Table S1).

**Table 1.** Winter Demographics and Measurements for All Participants and SER Groups

	All Participants (*n* = 35) (M/F, 19/16)		Emmetropia (*n* = 11) (M/F, 6/5)		Hyperopia (*n* = 13) (M/F, 4/9)		Myopia (*n* = 11) (M/F, 9/2)		
Parameter	Mean	SD	Mean	SD	Mean	SD	Mean	SD	*P* *
Age, y	19.83	2.2	20.09	1.92	18.54	1.9	21.09	2.12	<0.012
AL, mm	23.83	1.35	23.31	1.00	23.22	0.91	25.06	1.20	<0.001^*^
Cycloplegic SER, D	−0.50	2.58	−0.06	0.28	1.36	1.46	−3.14	2.61	<0.001^*^

AL mean is the group average from the individual average of all measurements at day 8.

* *P* values are by SER group and were adjusted by applying the Holm–Bonferroni method.

Table 2 shows the winter baseline and summer measurements for the 24 participants who were evaluated both seasons. Nine participants had hyperopia, six had emmetropia, and nine had myopia. Mean AL increased significantly from winter to summer (*P* = 0.003). Morning MEL (HWT+1) did not vary across seasons, but evening MEL (HST+0) was significantly higher in the summer (*P* = 0.001). There was no season by SER group effect for MEL measurements (Supplementary Table S2). Mean central corneal thickness and corneal radius exhibited negligible changes from winter to summer (2.4 µm and 0.01 mm, respectively) and are not discussed further in this paper.

**Table 2.** Seasonal Comparison for the 24 Participants (13 Males) Who Participated in Winter and Summer

	Winter		Summer		
Parameter	Mean	SD	Mean	SD	*P* Value by Season*
Age, y	19.58	2.39	20.04	2.56	<0.001
AL, mm	23.91	1.4	23.96	1.40	0.001
Morning MEL, pg/mL	12.07	10.25	9.35	8.92	0.120
Evening MEL, pg/mL	16.14	7.92	22.64	7.24	< 0.001

Mean values for AL and MEL are the group average from the individual average calculated for each respective season, except for morning MEL and evening MEL, which are the group average of MEL 1 hour after habitual wake-up time (HWT+1) and at habitual sleep time (HST+0), respectively.

* *P* values were adjusted by applying the Holm–Bonferroni method.

### Diurnality of Ocular Parameters and MEL: Seasonal Comparisons

**Figure 3.**
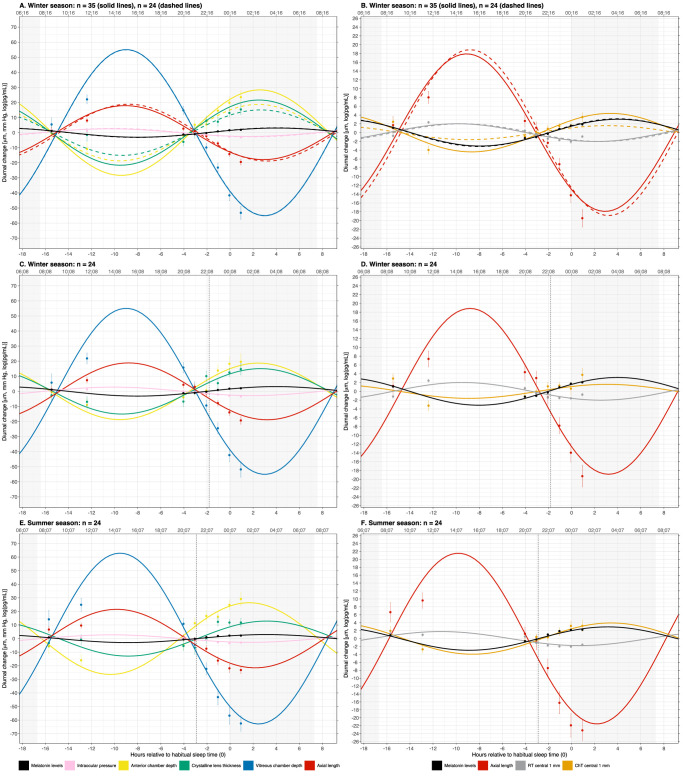
Group averages (±SE *error bars*) for measured ocular parameters or MEL, normalized to each individual's MESOR to emphasize relative differences. *Solid lines* show the sinusoidal model fits from the population estimate based on the fixed-effect estimates from the NLME model. The *gray* *areas* represent the averaged estimated sleep period. The *x*-axis is relative to habitual sleep time, where 0 is HST (the upper *x*-axis shows standard clock time for easier comparison with other studies). (**A, B**) Fitted models for all 35 participants in the winter study (*solid lines*) and for the 24 participants who returned for summer testing (*dashed lines*) showing that the latter are representative of the former group. **A** shows MEL, IOP, ACD, LT, VCD, and AL; **B** shows AL again (for reference), MEL, RT, and ChT with a rescaled *y*-axis for clarity. (**C, D**) The dashed sinusoids from **A** and **B** reproduced as *solid lines* for easier comparison to **E** and **F**. (**E, F**) Sinusoidal fits for the 24 participants in the summer study, to be compared directly with the same participants’ winter data in **C** and **D**. In **C**, **D**, **E**, and **F,** the *dashed vertical line* indicates the timings of DLMO relative to HST. The larger amplitude in VCD compared with AL is mainly accounted for by the antiphase changes in ACD, LT, and ChT.

Figure 3 shows the diurnal variations of the different ocular parameters and melatonin including the sinusoidal function fitted to the data for each season (each *y*-axis in the right panels is rescaled for clarity). Figures 3A and 3B show comparisons between the 35 winter-only participants versus the 24 winter and summer participants. There was no difference between the 24 participants when comparing them with the remaining 11 for any ocular parameter or MEL (*P* > 0.05 for all), indicating that the 24 participants who returned for the summer measurements were representative of the original 35 participants. Figures 3C to 3F show the data for the 24 participants for winter (Figs. 3C, 3D) and summer (Figs. 3E, 3F). Melatonin, IOP, ACD, LT, VCD, AL, RT, and ChT exhibited significant diurnal variation in each season (all *P* ≤ 0.002) (Table 3). The near in-phase relationship between MEL and LT, ACD, and ChT and the near 12-hour antiphase relationship between MEL and IOP, AL, VCD, and RT are apparent (Fig. 3).

**Table 3.** Linear Mixed-Effects Model Results Used to Determine Significant Diurnal Variations in the Ocular Parameters and MEL

	Winter (*n* = 24)		Summer (*n* = 24)	
Parameter	*F* Statistic	*P*	*F* Statistic	*P*
MEL	49.13	<0.001	48.30	<0.001
IOP	61.68	<0.001	45.72	<0.001
ACD	9.37	<0.001	13.74	<0.001
LT	5.04	<0.001	4.99	<0.001
VCD	61.01	<0.001	40.74	<0.001
AL	32.67	<0.001	27.88	<0.001
RT central 1 mm	17.56	<0.001	10.22	<0.001
ChT central 1 mm	6.65	<0.001	3.48	<0.002

HST occurred at the same standard clock time in winter as summer; thus, the diurnal characteristics of the ocular parameters are listed relative to HST in Table 4, with acrophases repeated in standard clock time in Table 5. DLMO, similarly to MEL acrophase, occurred nearly 1 hour earlier in the summer than in the winter. A similar phase advance was observed for ACD and AL, whereas the phase advance for VCD and IOP was smaller. A relative minor phase delay was observed for LT and ChT. As a result, in summer, ACD maintained its relationship with MEL, whereas IOP, LT, VCD, and ChT became more in phase with MEL and AL became more antiphase with MEL. There was a significant change in the phase relationship between AL and ChT, which moved from exact antiphase in the winter to being nearly antiphase in summer: 12.1-hour versus 10.7-hour difference in acrophase (*t*_23_ = 13, *P* < 0.001) (Table 4).

**Table 4.** Population Estimates for MESOR, Amplitude, and Acrophase for Ocular Parameters, and MEL for Winter and Summer Calculated from the NLME Model

	Winter						Summer					
	MESOR		Amplitude		Acrophase, h		MESOR		Amplitude		Acrophase, h	
Parameter	Est.	95% CI	Est.	95% CI	Est.	95% CI	Est.	95% CI	Est.	95% CI	Est.	95% CI
MEL, log(pg/mL)	0.87	0.66–1.07	3.155	2.92–3.39	3.99	3.62–4.30	1.04	0.82–1.25	2.952	2.71–3.2	3.14*	2.67–3.54
IOP, mm Hg	13.36	12.24–14.47	2.810	2.38–3.32	−10.10	−10.62 to −9.57	13.09	11.89–14.3	2.760	2.33–3.29	−10.27	−10.8 to −9.72
ACD, mm	3.73	3.62–3.84	0.019	0.011–0.028	2.44	1.82–2.99	3.74*	3.63–3.84	0.026	0.019–0.035	1.64	1.04–2.19
LT, mm	3.55	3.46–3.65	0.015	0.01–0.022	2.64	1.53–3.51	3.56	3.47–3.65	0.013	0.008–0.021	3.18	1.98–4.05
VCD, mm	16.41	15.85–16.97	0.055	0.048–0.063	−9.00	−9.43 to −8.55	16.44*	15.88–17	0.063	0.053–0.074	−9.53*	−9.91 to −9.15
AL, mm	23.92	23.37–24.47	0.019	0.015–0.024	−8.79	−9.24 to −8.34	23.96*	23.41–24.52	0.022	0.017–0.028	−9.81*	−10.24 to −9.37
RT central 1 mm, µm	261	256–267	2	1–3	−9.47	−10.30 to −8.60	263*	257–268	2	1–2	−11.32*	−12.52 to −9.97
ChT central 1 mm, µm	354	310–397	2	1–3	3.10	2.42–3.66	343*	302–385	4*	2–7	3.42	2.96–3.82

Acrophase is relative to habitual sleep time.

* Significant seasonal effect on MESOR, amplitude, or acrophase set at *P* < 0.05.

**Table 5.** Relative Acrophase From Estimates Given in Table 4 Converted to Standard Clock Time in Winter and Summer

	Acrophase				
	Winter		Summer		
Parameter	Standard Clock Time	95% CI	Standard Clock Time	95% CI	Seasonal Change
ACD	02:34	01:57–03:07	01:46	01:10–02:19	−00:48
LT	02:46	01:40–03:38	03:02	02:01–04:10	+00:16
ChT central 1 mm	03:14	02:33–03:47	03:32	03:05–03:56	+00:18
MEL	04:07	03:45–04:26	03:16	02:47–03:40	−00:51
IOP	14:02	13:31–14:34	13:51	13:19–14:25	−00:11
RT central 1 mm	14:40	13:50–15:32	12:49	11:37–14:09	−01:51
VCD	15:08	14:42–15:35	14:35	14:13–14:58	−00:33
AL	15:20	14:53–15:47	14:19	13:53–14:45	−01:01
DLMO	22:20	21:55–22:44	21:17	20:53–21:42	−00:47

The – and + signs indicate phase advance and delay, respectively. DLMO (not an acrophase) is included for comparison.

### Seasonal Changes in Diurnal Characteristics and Phase Relationships

**Figure 4.**
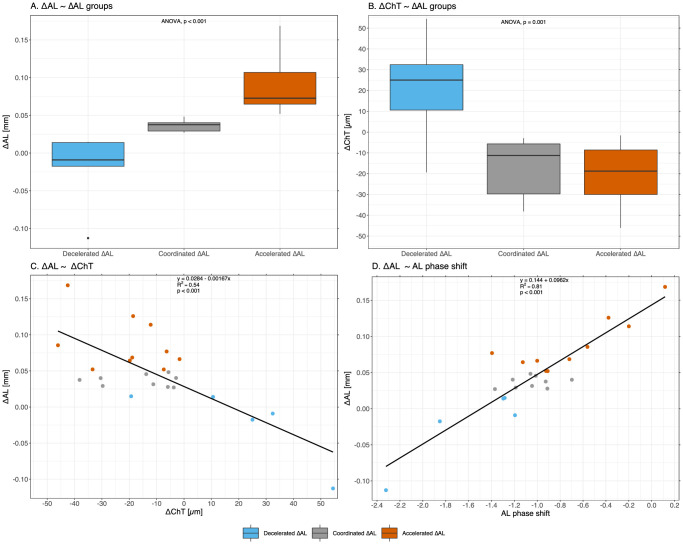
Associations between seasonal change in AL (∆AL and AL phase shift) and seasonal change in choroidal thickness (∆ChT). Participants have been grouped according to the rate of ∆AL—decelerated ∆AL (*blue*), coordinated ∆AL (*gray*), and accelerated ∆AL (*red*)—using definitions of ∆AL based on expected ∆AL after 7 months^15^ (see text for details). (**A**) Boxplot of AL seasonal change for the ∆AL groups. (**B**) Boxplot of ∆ChT for the ∆AL groups. (**C**) Scatterplot and linear regression (*solid line*) of ∆AL as a function of ∆ChT. (**D**) Scatterplot and linear regression (*solid line*) of ∆AL as a function of AL phase shift. Significance at *P* < 0.05 for one-way ANOVA.

There was a significant change in MESOR from winter to summer for several ocular parameters: deeper ACD and VCD, longer AL, thickening of the retina, and thinning of the choroid (Table 4). There was also a significant increase in amplitude for ChT from winter to summer. To assess the association between change in AL (ΔAL) and ChT (ΔChT) from winter to summer, we divided the participants into three groups based on previous reports of expected ΔAL when there was coordinated ocular growth (axial elongation without a change in refractive error) over a 2-year period at the same latitude^13^ and calculated the expected ΔAL for the winter to summer period (7 months). Nine participants had an increase in AL that was deemed to be coordinated growth (0.017 mm ≤ ΔAL < 0.052 mm), 10 participants had an increase in AL that was more than expected from coordinated growth (accelerated ΔAL = 0.052–0.169 mm), and five participants had a minor increase or a decrease in AL that was less than expected from coordinated growth (decelerated ΔAL = −0.113–0.017 mm). Fisher's exact test revealed that the SER groups and the three ΔAL groups were independent from each other (*P* = 0.7). Figure 4A shows that there was a significant difference among these three ΔAL groups (*F*_2,__21_ = 17.8; *P* < 0.001; all *P* < 0.015, Tukey's honestly significant difference [HSD] test). Figures 4B to 4D show that ΔChT (MESOR) across seasons differed significantly among the groups; ChT increased in the group with decelerated ΔAL, whereas it decreased in the two other groups (*F*_2,__21_ = 9.83; *P* = 0.001; all *P* < 0.004, Tukey's HSD test). Figure 4C show that ΔAL was not fully accounted for by ΔChT (*F*_1,__22_ = 25.7; *P* < 0.001; *R*^2^ = 0.54).

Figure 4D shows the strong association between ΔAL and AL phase shift. The group with decelerated ∆AL had a significantly larger phase advance for both VCD and AL across seasons (*F*_2,21_ = 5.28; *P* = 0.014; *P* = 0.011, Tukey's HSD test) compared with the group with accelerated ∆AL (*F*_2,__21_ = 8.68; *P* = 0.0018; *P* = 0.001, Tukey's HSD test). There were no differences between the other group combinations. In summer, there was a significant difference between the decelerated ∆AL group and the accelerated ∆AL group for RT (*F*_2,21_ = 3.76; *P* = 0.04; *P* = 0.032, Tukey's HSD test). There were no other differences of MESOR, acrophase, or amplitude for other ocular parameters, evening, morning, or mean MEL nor DLMO between ΔAL groups nor SER groups. There were no associations between chronotype, neither MEL acrophase nor DLMO, and ΔAL or SER (Supplementary Table S3).

### Annual Versus Seasonal Changes in AL

For the 10 participants who returned the following winter, we assumed there were no differences in HST between the first winter (W1) and the second winter (W2). The W2 measurements did not occur at the same time (in standard time, local clock time, or habitual sleep time) as the W1 times, so the former were re-expressed relative to the individual's habitual sleep time and used to derive AL values for the W1 and summer (S) seasons, using their respective sinusoid models. Using time-matched AL measurements in this way minimized any influence of diurnal changes arising from differences in measurement times between the three seasons.

As for the original 24 participants, there was a significant increase in mean AL from winter to summer for these 10 participants (pairwise comparison *F*_1,__9_ = 20.90; *P* = 0.001). There was no change in mean AL from S to W2 (pairwise comparison *F*_1,__9_ = 0.028; *P* = 0.87); however, from W1 to W2 there was significant annual change in mean AL (pairwise comparison, *F*_1,__9_ = 6.26; *P* = 0.034). Nine of the participants had a positive annual change in AL. To assess the degree of annual change in AL we calculated expected ∆AL over 12 months and grouped participants in the same manner as described above. Table 6 shows that the annual change (W1 to W2) in AL differed among participants. Eight participants (Table 6, participants 2–9) had seasonal variation in ∆AL, with an increase in AL from W1 to S followed by a smaller increase or, for some, a decrease, in AL from S to W2. Two participants had a change that indicated no growth (0.000 < ΔAL ≤ 0.030 mm); five, coordinated growth (0.030 < ΔAL ≤ 0.089 mm); and one, accelerated growth (∆AL > 0.089 mm). Focusing on those who had a change in SER ≤  –0.50 (which is more than the actual range of repeatability for the worst case, 0.36 D, for the HRK-8000A), we see that participant 7 (who had low hyperopia at baseline and had seasonal variation in axial elongation) exhibited an annual elongation that led to a more myopic SER (∆SER = −0.54). Participant 10, a myope at baseline, had equal axial elongation across seasons and the largest change in SER of all participants (∆SER = −0.58). Participant 1 had no change from W1 to S followed by a decrease in AL from S to W2, indicating AL shortening (∆AL = –0.062 mm) with no meaningful change in SER. As was shown for the original 24 participants, there was no significant difference in mean ∆AL between SER groups from W1 to S for these 10 participants either, from neither S to W2 nor W1 to W2.

**Table 6.** Baseline and Annual Change in SER and AL

	AL, mm	∆AL, mm			SER, D	∆SER, D
Participant	W1	W1–S	S–W2	W1–W2	W1	W1–W2
1	22.427	−0.011	−0.051	−0.062	−0.48	−0.17
2	22.968	0.068	−0.045	0.023	−0.23	−0.18
3	25.003	0.050	−0.023	0.027	0.22	−0.14
4	23.596	0.021	0.010	0.031	−0.91	−0.22
5	23.746	0.044	−0.009	0.035	1.06	−0.19
6	24.892	0.067	−0.015	0.052	0.20	−0.05
7	23.325	0.049	0.010	0.059	0.67	−0.54
8	21.938	0.046	0.022	0.068	0.25	−0.25
9	24.898	0.125	0.020	0.145	−4.08	−0.04
10	24.879	0.110	0.105	0.215	−0.92	−0.58

∆AL values are from winter (W1) to summer (S), summer to the second winter (W2), and W1 to W2 (annual change). ∆SER values are from W1 to W2. Table is ordered by increasing ∆AL from W1 to W2.

## Discussion

In this study of young adults living at latitude 60°N, seasonal variation in the diurnal rhythms of melatonin secretion and ocular parameters were observed. Specifically, AL exhibited an earlier acrophase in the summer compared with winter. This followed suit with MEL, with an earlier DLMO and earlier acrophase in the summer, as reported by others.^58^^–^^60^ Choroidal thickness exhibited a slight delay in acrophase, resulting in a significant seasonal shift in the relationship between AL and ChT.

The reported 1-hour phase advance in summer for MEL^60^ and DLMO,^58^ also observed in this study, coincided with an earlier acrophase for VCD and AL (Table 5). This alludes to the suggestion that these are synchronized to a master clock (that changes with season) and that our methods are sensitive enough to detect this. A previous study conducted in northeast Australia failed to observe seasonal variation in diurnal rhythms of ocular structures, in either AL or any other ocular structure.^31^ This could be due to the considerably smaller seasonal difference between the two study locations, with only 2 hours (northeast Australia) more daylight in the summer than winter^28^ (c.f. 12 hours in southeast Norway). In other chronobiology studies, a seasonal shift is attributed to the increased number of daylight hours in the summer combined with changes in social behavior (e.g., spending more time outdoors when it is warmer) and daylight saving time.^27^^,^^58^^,^^60^ Seasonal variation of ocular diurnal rhythms may therefore only be observable at latitudes with sufficient differences in number of daylight hours between seasons.^61^

The observed diurnal variation of measured ocular structures supports previous findings.^3^^–^^6^ Here, we show that changes in AL are significantly associated with changes in the phase relationships between AL and ChT across seasons (Fig. 4D). In the 7 months between the measurements in winter and summer, both increases and decreases in AL were observed, with 22 of 24 participants having an AL change that was larger than the reported repeatability limit (±0.014 mm) for the IOLMaster 700.^37^ Interestingly, eyes that were measured to be shorter had a larger phase-advance of AL and a thickening of the choroid, whilst the opposite was observed in eyes that were measured to be longer (Fig. 4D). The association between a near antiphase (rather than exact antiphase) relationship of AL and ChT and AL shortening and ChT thickening is a novel finding in humans. It is analogous to experimental animal models where a near antiphase (rather than exact antiphase) relationship between AL and ChT has been associated with normal ocular growth.^9^^,^^10^

Experimental studies with both animals^62^^,^^63^ and humans^21^ indicate that increased ambient light exposure can result in choroidal thickening. Exposure to daylight has been implicated to play a protective role in ocular growth in children,^19^^,^^64^ with less growth during summer compared with winter.^29^^,^^65^ ^66^ This growth pattern was also evident in six of the participants who returned the following winter and who were deemed to have maintained their refractive error in the 12 months between the first and the third set of measurements. Five had increases in AL that were compatible with coordinated growth (0.023 mm ≤ ΔAL ≤ 0.052 mm) (Table 6, participants 2–6), and one presented with annual AL shortening (ΔAL = −0.062). The decrease in AL in either season observed in some participants and throughout the year for this one participant could be indicative of axial elongation being reversible—as previously reported in animals^67^ and humans (children,^68^^–^^72^ as well as adolescents and young adults^13^^,^^31^^,^^73^^,^^74^). Nonetheless, as reported from animal studies,^67^ the amount of choroidal thickening does not equal the amount of eye shortening. Here, only 54% of the variation in AL was explained by the change in ChT (Fig. 4C); thus, further research is needed to understand what AL shortening entails.

Daylight exposure has also been associated with the size of decrease in AL measured at midday and then again in the evening in humans, with less daylight exposure being associated with a longer AL at midday and an associated larger decrease in AL and more AL elongation.^31^ We did not find such differences; that is, AL amplitude was not associated with ∆AL regardless of season or grouping. This is reminiscent of findings in chicks.^22^^,^^75^ Furthermore, there was no association between AL MESOR and AL amplitude for either season. It would not be unreasonable to assume that our participants’ exposure to daylight was considerably lower during the autumn–winter period with few available daylight hours compared with the spring–summer period at this latitude. Unlike other studies,^31^^,^^76^ we did not measure the participants’ daylight exposure. In the Australian study (mentioned above), they did not control for their participants’ habitual sleep times, ignoring their individual chronotypes, which could mean that they incorrectly calculated average epochal measurements from different points in each individual's rhythm. Moreover, their only evening measures were at 18:00 and 21:00 and, consequently, may have missed their participants’ true AL minima. There is also the possibility that our participants are susceptible to other myopigenic factors.^77^

Neither habitual sleep time nor single measures of MEL (such as DLMO) differed among the SER groups or ∆AL groups. Although the phase of MEL did not differ among the SER groups (as reported by others),^78^ we also failed to find a significant association between MEL concentration and SER. Similarly, neither we nor others^5^ have found that circadian rhythms of ocular parameters differed among refractive error groups. Furthermore (and in line with reporting standards in chronobiology studies),^36^^,^^79^ our results indicate that the relationship between MEL and SER or AL alone is not sufficient to explain the influence of circadian synchronization on ocular growth. Instead, it must be combined with knowledge about the changes in phase relationships among ocular parameters.

The diurnal rhythm and acrophase of ACD was nearly 3 hours past HST (Table 5), in agreement with most studies.^3^^–^^5^ Contrary to existing studies,^4^^–^^6^ however, we found that the diurnal rhythms of ACD and LT were in phase, increasing in depth and thickness throughout the evening with maximum diurnal changes of +38 and +30 µm, respectively (Table 4, Fig. 3). This behavior differs from accommodation, where an increase in lens thickness (i.e., increase in the curvature of the anterior and posterior surfaces of the crystalline lens) leads to a shallower ACD, with the anterior lens surface accounting for most of the LT change.^80^^,^^81^ An accommodative stimulus of 3 D has been reported to alter LT by +138 µm and, subsequently, ACD by –106 µm.^82^ The most parsimonious explanation is that the diurnal changes of the crystalline lens primarily occur at the posterior surface,^4^ which is compatible with the diurnal rhythm of VCD being in antiphase to LT. These changes differ from the biomechanical changes associated with accommodation and are substantiated by the in-phase diurnal rhythms of LT and MEL (in both winter and summer) (Fig. 3).

It is known that there are large between-individual variations in foveal shape^83^ and metrics,^84^ but neither has been found to correlate with refractive error or AL.^85^ The diurnal rhythm of central RT was not associated with SER or AL and appears to be like that reported previously.^5^ That the advance in acrophase in RT is nearly an hour more than MEL and the other measures is a novel finding, and more work is needed to understand this.

### Strengths and Limitations

There are three key strengths to the methodology presented here. First, all measurement epochs were relative to the individual's habitual sleep time (measured objectively with actigraphy). The results imply that rhythmic changes in melatonin and ocular parameters are regulated by an internal bodily mechanism, and making measurements relative to that internal mechanism has demonstrably delivered robust measurements of those rhythms. In contrast, studies where measurements were made relative to wall-clock time^3^^,^^4^^,^^31^ may not yield meaningful population measures unless the population was carefully selected to have the same wake–sleep cycle as one another.^5^ Second, the per-individual timing of the epochs encompassed a full waking day—from 1 hour after habitual wake time to 1 hour after habitual sleep time—and thus minimally impacted the participant's normal rhythms. Such a wide range of measurements improves model fitting when data samples are collected at time points spanning both the peaks and troughs of the fitted sinusoids. Other studies have reported ACD,^5^ LT, and RT^4^ not exhibiting diurnal variation or LT barely reaching significance.^6^ These variations may be explained by the different measurement intervals varying from six times every 3 to 7 hours^3^ or five times every 2.5 to 3 hours.^4^ Even a regimented and exhaustive measurement routine (every 4 hours for 24 hours^5^^,^^6^) would involve disturbing participants’ sleep patterns and, consequently, their ocular biometry measurements. On a related matter, fitting actual sinusoidal functions to the data and utilizing the MESOR (rather than the arithmetic mean) of the data points also improved the robustness of the results, as the MESOR is not biased by the timing of the measurements. Third, our data collection methodology controlled or nullified as many confounding variables as possible during data collection, including strict exclusion criteria for visual function when selecting participants; carefully controlled lighting conditions during and between measurement epochs; ensuring that the participants remained seated throughout all epochs (to avoid possible effects of postural changes on measurements)^34^^–^^36^; and preventing participants from engaging in any near-distance tasks or exposure to bright light sources between measurement epochs.

A limitation of our study is the small number of participants and that we measured the participants on weekdays only and not during a weekend; sleep behavior has been found to vary throughout the weekend, when DLMO can be delayed.^86^ In terms of daily variations of ocular parameters, however, a previous study found no differences in AL and ChT between weekdays and weekends.^31^ Another limitation was that we did not measure participants’ light exposure. Ambient light levels were controlled for in the laboratory 15 to 20 minutes before the first measurement session and throughout the remaining evening; however, we do not know the amount of light the participants were exposed to before arriving at the laboratory. At the latitude studied here, there are considerable differences in daylight availability and intensity between winter and summer.^27^ Whether it is the duration of daylight exposure or its intensity or temperature, or a combination thereof, that is important for ocular growth requires further study.^27^ Regrettably, only 10 of the original 24 participants were available for a measurement at W2 which limits the robustness of the annual analysis. Including them, however, emphasizes the importance of understanding annual variations in eye growth among individuals.

## Conclusions

We report significant seasonal variation in the diurnal rhythms of MEL, VCD, and AL. These rhythms appear to be synchronized in both winter and summer, suggesting that the overall rhythm of the eye expressed through ocular AL is synchronized with the master circadian clock, as measured by saliva melatonin secretion. Additionally, the direction and degree of AL change were significantly associated with the degree of AL phase advance in the summer, alluding to between-individual variation of the eye in adaptation to seasonal changes in diurnal rhythms (at least at latitudes where there are notable seasonal changes in daylight exposure). The finding that the crystalline lens and anterior chamber underwent significant in-phase diurnal variations, both increasing in size in the evening, renews interest in the role that time-of-day exposure of near-distance activities may play in eye growth.^87^ It indicates that other behavioral modifications, in combination with daylight exposure, may contribute to delay myopia onset and development.

## Supplementary Material

Supplement 1
