# Supplementary material for: Seasonal Variation in Diurnal Rhythms of the Human Eye: Implications for Continuing Ocular Growth in Adolescents and Young Adults
Source: Invest Ophthalmol Vis Sci. 2022 Oct 25;63(11):20. doi: 10.1167/iovs.63.11.20 (PMC9617503; doi:10.1167/iovs.63.11.20)
Supplement: Supplement 1 [file iovs-63-11-20_s001.pdf]

Supplementary material

Seasonal variation in diurnal rhythms of the human eye: implications for continuing ocular growth in adolescents and young adults.

Nickolai G. Nilsen[1]

Stuart J. Gilson[1]

Hilde R. Pedersen[1]

Lene A. Hagen[1]

Kenneth Knoblauch[1,2,3]

Rigmor C. Baraas[1]

[1]National Centre for Optics, Vision and Eye Care, Faculty of Health and Social Sciences, University of South-Eastern Norway, Kongsberg, Norway

[2]Stem Cell and Brain Research Institute, INSERM U1208, 18 Avenue Doyen Lépine, Bron, 69500, France

[3]Université de Lyon, Université Lyon I, Lyon, 69003, France

Table S1. Statistics for table 1. One-way ANOVA. Tukey HSD was used to assess the differences between the SER groups. N = 35.

Model parameters	F (2, 32)	P-value	Hyperopia-emmetropia	Myopia-emmetropia	Myopia-hyperopia
Age ~ SER group	5.11	0.012	0.150	0.470	0.010*
AL ~ SER group	10.50	<0.000*	0.982	0.002*	0.001*
Cyclo SER ~ SER group	19.34	<0.000*	0.145	0.001*	<0.000*

Table S2. Statistics for table 2. Repeated measures ANOVA with season as within factor and SER group as between factor. AL mean is the group average from the individual average of all measurements at day 8. For morning MEL and evening MEL, the group average from single time points were used. N=24.

**Dependent variable**	**Within factor, between factor**	**F (DFn, DFd) Season**	**P-value Season**	**F (DFn, DFd) SER group**	**P-value SER group**	**F (DFn, DFd) Season by SER group**	**P-value Season by SER group**
Age	Season, SER group	16.62 (1, 21)	0.002*	8.48 (2, 21)	0.004*	0.33 (2, 21)	0.724
AL	Season, SER group	14.63 (1, 21)	0.003*	6.78 (2,12)	0.010*	0.30 (2, 21)	0.740
Morning MEL (HWT+1)	Season, SER group	2.49 (1, 21)	0.260	2.92 (2, 21)	0.228	2.11 (2, 21)	0.260
Evening MEL (HST+0)	Season, SER group	18.58 (1, 21)	0.001*	1.50 (2, 21)	0.494	0.25 (2, 21)	0.781
DLMO	Season, SER group	72.24 (1, 21)	<0.000	1.095 (2, 21)	0.353	2.097 (2, 21)	0.296

Tukey HSD post-hoc test showed that for both winter and summer, myopes were significantly older than hyperopes (p = 0.002). There were no differences between myopes and emmetropes (p = 0.130) nor between hyperopes and emmetropes (p = 0.308). In terms of AL, myopes had a longer AL than emmetropes and hyperopes (p = 0.035 and p = 0.006, respectively), there were no differences between emmetropes and hyperopes (p = 0.914)

Table S3. Associations between chronotypes and 1) ΔAL from winter to summer and 2) SER. Sleep onset, MEL acrophase and DLMO was analyzed using standard time. N=24.

Model parameters	F (3, 44)	R^2	p-value
ΔAL ~ DLMO * season	1.77	0.11	0.166
ΔAL ~ MEL acrophase * season	2.10	0.13	0.114
ΔAL ~ sleep onset * season	1.95	0.12	0.136
SER ~ DLMO2 * season	0.75	0.05	0.530
SER ~ MEL * season	0.66	0.04	0.578
SER ~ HST * season	0.72	0.05	0.544
